# A Sustainable Treatment Protocol for Organophosphate Poisoning in Rural Kenya for Facilities without Intensive Care Units and Where Transfer Is Not Possible

**DOI:** 10.4269/ajtmh.25-0266

**Published:** 2026-07-16

**Authors:** Jerome Koleski, Sommer Aldulaimi, Jonathan Nthusi

**Affiliations:** ^1^ Global Health Programs, College of Medicine, University of Arizona, Tucson, Arizona, USA;; ^2^ Department of Family Medicine, School of Medicine and Health Sciences, Kabarak University, Nakuru, Kenya

## Abstract

Organophosphate poisoning is a common method of attempting suicide in developing countries, particularly in rural areas. The current WHO protocols emphasize intubation and transfer to a facility with intensive care. Both recommendations are beyond the resources of many rural hospitals in developing countries and their poor patients. Because organophosphate poisoning is a commonly seen condition in Kenya and because intubation is beyond the equipment and training of many rural hospitals, a protocol involving atropine without intubation was developed and used. The protocol includes placement of a nasogastric tube, activated charcoal, intravenous fluids, and atropine along with other supportive measures; the protocol was developed based on a literature search of international medical journals and has been in use for years at Kapsowar Mission Hospital (KMH). Organophosphate poisoning outcome data at KMH were reviewed for 2021–2023. One patient died within minutes of presentation to the Casualty Ward, but the rest survived to discharge using the above protocol. The cost of the atropine protocol is over U.S. $200 in Kenya. The recommendations of the WHO are usually helpful to practitioners in underresourced facilities. In the case of organophosphate poisoning, the recommendations are more appropriate to higher-income countries and highest-level hospitals in low- and middle-income countries, facilities that have intubation capability and large quantities of atropine. A protocol using less atropine could be offered as an alternative.

## INTRODUCTION

Organophosphate poisoning accounts for approximately 12–17% of suicide attempts and suicide deaths worldwide and is responsible for up to one-third of suicides in rural Asia, where access to highly toxic pesticides is common.^1^ Another source estimates that organophosphate poisonings were responsible for more than 5,000,000 deaths in the three decades between 1986 and 2016.^2^ Many farmers in rural areas of Kenya spray their herds every 1 to 2 weeks with organophosphate to prevent loss of the major source of their wealth (their cows, sheep, and goats) to insect-borne diseases.^3^ Many LMICs do not have restrictions on the most toxic pesticides, and they are readily available throughout LMICs.^4^ Estimates are that one third of suicides each year worldwide are because of organophosphate.^5^

The WHO regularly publishes treatment protocols useful for rural practitioners. The WHO suggested protocol involves intubation, mechanical ventilation, and transfer to a facility with a higher level of care.^6^

At Kapsowar Mission Hospital (KMH) in western Kenya, organophosphate poisoning is a commonly encountered medical issue, yet intubation is not possible as the hospital lacks intensive care unit (ICU) capabilities and ventilators. In Kenya, only 22 of 47 counties have even one ICU,^7^ with 79% of ICU beds concentrated in the five largest cities.^8^ Many patients cannot afford a transfer to a facility that does have an ICU, or they may not be stable enough to survive until reaching the higher-level hospital. Therefore, KMH created a protocol that involves the use of atropine and isotonic intravenous fluids (IVFs).^9^ The mechanism of atropine specifically antagonizes acetylcholine muscarinic receptors that are affected by organophosphate.^7^ Many protocols emphasize doubling doses of atropine until parameters for pulse and blood pressure are met (Appendix 1).^7^^,^^10^^–^^12^ At KMH, this dose doubling exhausted the limited supplies of atropine; thus, the physicians at KMH adjusted the protocol to reduce atropine usage while maintaining supplies and keeping patients alive (Appendix 2).

With protocol guidelines already in place, we performed a literature search of international medical journals, and then, we refined the protocol guidelines in response to patient outcomes. Some protocols suggest doubling the dose of atropine with each administration.^2^^,^^11^ Because of lack of availability of large quantities of atropine used in protocols that call for doubling the dose of atropine at every administration, the physicians at KMH decided that 1 mg atropine per dose was more sustainable. Attempts at doubling the atropine dose led to rapid depletion of the atropine stores in the pharmacy, leading to stock outs and resulting in no atropine available for some patients.

Once organophosphate poisoning was identified by a history reported by the patient or family members, a nasogastric tube (NGT) was placed, gastric lavage was performed, activated charcoal was placed through an NGT, and a Foley catheter was placed. Isotonic IVFs, either lactated Ringer’s or normal saline, were given at rates up to 200 mL/hour to a target systolic blood pressure (SBP) greater than 80 mm Hg. Atropine was given in 1-mg increments initially and up to every 15 minutes to maintain SBP >80 mm Hg and pulse >80 beats per minute. These measures were targets as at times, it was only possible with the doses of atropine available to reach SBP >60 mm Hg and pulse >60 beats per minute until the body had cleared more of the organophosphate.

Atropine at 1 mg every 15 minutes and IVFs at 200 mL/hour were the maximum dosing used in the protocol. Atropine was not dosed higher because of the risk of exhausting the hospital’s limited supply of atropine. If the Glasgow Coma Scale (GCS) (Appendix 3)^12^ was less than 10, the NGT was left in place, and the patient was placed in a decubitus position (side lying). A Foley catheter was placed in all patients until they were able to ambulate to the toilet with assistance.

Once the SBP went higher than 80 mm Hg or the pulse was >80 beats per minute, the time interval between atropine doses was increased from 15 to 30 minutes and then, to 60 minutes. Often, the patients would need atropine 1 mg intravenously every 4 to 6 hours until 72 hours after admission.

Some protocols in the literature recommend doubling the atropine dose with each administration.^11^ This exponential doubling was not practical or sustainable at KMH as the limited supplies of atropine were used up too quickly. It was decided that using only 1 mg of atropine per dose was the most sustainable option in that resource-limited setting.

Simple maneuvers to determine the extent of residual organophosphate poisoning were daily evaluation of the GCS and testing the patient’s orientation to person, place, and time. Once the patient had a GCS of 15, including being oriented to person, place, and time for 24 hours and able to ambulate with minimal assistance, the patient and the family were interviewed by the Chaplaincy Service and the physicians to determine continued risk of another suicide attempt. Although a psychiatric or psychologic evaluation would have been better, those specialties were not available at KMH. If the patient met all of the above criteria, the patient was deemed stable for discharge (Appendix 2).

## MATERIALS AND METHODS

A retrospective chart review was accomplished by pulling all of the records with the International Classification of Diseases, Tenth Revision (ICD-10) codes T60.0X1 and T60.0X1A (toxic effect of organophosphate and carbamate insecticides unintentional and accidental, respectively) for the dates January 1, 2021 through December 31, 2023. All of the records were compiled into an Excel spreadsheet (Microsoft Excel, Microsoft Corp, Redmond, WA) that deidentified the patient, listing the age of each patient, sex, length of stay, condition upon discharge (alive or dead), and a good-faith effort to estimate the GCS based on the documentation of the admission history and a physical. If the progress note tracked the GCS, that was included as well (Appendix 3).

The cost of the protocol was calculated by taking the patient cost of atropine from the KMH pharmacy (100 Kenyan shilling = 0.77 U.S. dollar per 1 mg) and the patient cost of activated charcoal (15 KSH = 0.12 U.S. dollar per tablet). The dosage costs were then multiplied by the recommended doses over the course of the protocol. There is no specific charge for NGT at KMH.

The data collection protocol was reviewed by the administration of KMH as there is not a formal institutional review board at that facility, the Institutional Ethical Review Committee of Africa Gospel, the Church Tenwek Mission Hospital, and the Institutional Review Board of the University of Arizona College of Medicine.

## RESULTS

Table 1 has aggregated data for each year from 2021 to 2023.

**Table 1:** Statistics of Kapsowar Mission Hospital organophosphate poisoning patients for January 1, 2021 to December 31, 2023 by year and overall

Patient Information	2021			2022			2023			2021–2023		
	Total	Male	Female	Total	Male	Female	Total	Male	Female	Total	Male	Female
OPP admissions	33	20	13	21	14	7	21	17	4	75	51	24
Age range (years)	11–49	11–49	14–49	16–62	16–33	19–62	10–31	10–31	10–19	10–62	10–49	10–62
Median age (years)	20	20	20	25	25	50	18	18	15.5	20	20	20
Length of stay range (days)	1–7	1–7	1–5	0–7	1–7	0–3	1–7	1–7	1–5	0–7	1–7	0–5
Average stay (days)	2.4	2.6	2.2	2.4	2.6	2.0	2.4	2.4	2.5	2.4	2.5	2.2
Alive on discharge (%)	100	100	100	100	100	100	100	100	100	100	100	100
Average GCS on admission	12.0	11.4	12.8	11.9	11.6	12.5	12.3	12.0	14.0	12.1	11.7	12.9

GCS = Glasgow Coma Scale; OPP = organophosphate poisoning.

There were 75 patients admitted with organophosphate poisoning over the study period. The ratio of men to women varied from 1.5× in 2021 to 4× in 2023, but men always had a higher number than women.

The average GCS was lower in males, and men had longer average hospital stays. Patients of both sexes with a lower GCS had lower SBP and pulse, showed evidence of having required larger quantities of atropine and IVF to stabilize, and had longer hospital stays.

The average length of stay was 2.0 to 2.6 days across the years. The range of stay was 0 to 7 days, which was usually correlated with the amount of organophosphate consumed and inversely related to the GCS on admission.

The age range was from 10 to 62 years old. The median age for the 3 years from 2021 to 2023 was 20 years old for both men and women. In 2021, the median age was 20 years old for both men and women. In 2022, the median age was 25 years old for men and 50 years old for women. In 2023, the median age was 18 years old for men and 15.5 years old for women. The age range of the youngest organophosphate poisoning patients was 10 to 16 years old in the 3 years studied.

The survival rate of patients who received the atropine protocol was 100%. The survival rate indicates patients who survived to discharge after being formally admitted to the hospital. There was at least one patient who passed within less than 10 minutes of arrival to the Casualty Ward and therefore, was not admitted to either the hospital or the Casualty Ward; thus, he was not counted.

## DISCUSSION

It is logical that patients consuming more organophosphate would have lower GCS, have longer hospital stays, and need more atropine. Men are a much higher proportion of those with organophosphate poisoning. The survival rate to discharge of 100% shows that the 1-mg atropine up to every 15 minutes dosing regimen is a viable option for treatment of organophosphate poisoning where supplies of atropine are too limited to sustain the dose doubling scheme.

The wide variability in the median age of women admitted for organophosphate poisoning may be because of the lower numbers of women attempting suicide by organophosphate ingestion. With fewer numbers, the variability in median age will vary more widely from year to year. Other explanations for the wider variability of the median age of women patients are possible.

There are limitations to the study. First, more toxic organophosphates are banned in Kenya,^13^ meaning that less expensive, less equipment-dependent, and less atropine-consuming interventions still allow the body to clear the poison. This lower-dose protocol may be less effective in places where the class I more hazardous pesticides are allowed.^6^ Second, because this protocol had been used at KMH for many years, clinical staff were highly familiar with its implementation. Frequent monitoring of vital signs and close patient follow-up may have partially compensated for the lack of ICU-level care, allowing patients to be successfully managed with lower doses of atropine than those recommended in protocols that utilize dose doubling. Third, this study was a retrospective chart review, so there was no direct comparison, either case-controlled retrospective or prospective, with the doubling dose of atropine protocol.

The authors do not suggest undertreating organophosphate poisoning in facilities where intubation and ICUs are available, and this protocol does not suggest not following the standard doubling dosage scheme of atropine if such quantities are readily available at the facility in question. The protocol presented is meant as an aid to providers in settings where intubation, critical care, and larger quantities of atropine are unavailable.

The costs per dose may vary in other countries, and the strength of pesticides used may also vary. The most common pesticides used in Kenya are WHO class II.^14^

## CONCLUSION

Organophosphate poisoning, both deliberate and accidental, is a risk in rural areas of LMICs for the foreseeable future. This protocol is an alternative for more resource-limited facilities where transfer to higher-level facilities is not a readily available option.

## APPENDIX 1^9^^,^^10^

1. Initial phase (rapid atropinization)
  a. Administration. Sequential, doubling bolus doses were used.
  b. Goal. Dosing was adjusted until the desired effect was achieved.
2. Maintenance phase
  a. Starting infusion. An infusion was initiated at about 20% to 30% of the starting dose.
  b. Adjustment. The infusion rate was adjusted based on the observed effect.
3. Protocol adjustments
  a. When insufficient. The infusion was increased and combined with bolus doses if needed.
  b. If toxicity occurred. The infusion was stopped and restarted at a lower rate after toxicity resolved.

## APPENDIX 2

The Kapsowar Mission Hospital atropine protocol for organophosphate poisoning and costs in KSH and U.S. dollars.

The protocol followed at the Kapsowar Mission Hospital is as follows.

1. Placement of a nasogastric tube
2. Gastric lavage to remove any remnants of organophosphate in the stomach
3. Activated charcoal through a nasogastric tube
  a. 100 g = 50 g/tablet × 2 (15 KSH = 0.12 U.S. dollar per tablet).
4. Atropinization of the patient
  a. Atropine 1 mg intravenously every 15 minutes until the lungs are clear from audible respiratory secretions and
    i. (100 KSH = 0.77 U.S. dollar per 1 mg)
  b. two of the conditions are met
    i. systolic blood pressure >80 mm Hg
    ii. pulse >80 beats per minute
    iii. oral secretions are dry
5. Atropine is weaned from every 15 minutes to every 30 minutes, then to every hour, and finally, to every 4 to 6 hours until it is discontinued. The adjustments are based on systolic blood pressure, pulse, and respiratory secretion status.
6. (Although some protocols indicate doubling atropine amount with each dose [1, 2, 4, 8, etc.], this dosing scheme exhausted the limited supplies of atropine at the Kapsowar Mission Hospital pharmacy. Thus, it was decided that a sustainable dosing scheme was preferable to the “ideal dosing scheme.”)

## APPENDIX 3^12^

Glasgow Coma Scale

Best eye response

4. Eyes open spontaneously
3. Eyes opening to sound
2. Eyes opening to pain
1. No eye opening

Best verbal response

5. Orientated
4. Confused
3. Inappropriate words
2. Incomprehensible sounds
1. No verbal response

Best motor response

6. Obeys commands
5. Localizing pain
4. Withdrawal from pain
3. Abnormal flexion to pain
2. Abnormal extension with pain
1. No motor response

Maximum: 15; minimum: 3

With a Glasgow Coma Scale of eight or less, the patient is considered unable to protect his or her airway.
